# 

**Published:** 1847-04-01

**Authors:** 


### BIBLIOGRAPHICAL RECORD.

1. On the Relations of the Physician to the Sick, to the Public, and to his Colleagues. By the late *Christopher William Hufeland*, M.D. Pp. 41. Oxford & London.

2. Elements of Chemistry, including the actual State and prevalent Doctrines of the Science. By the late *Edward Turner*, M.D. 8th Edition. Edited by Baron *Liebig* and *William Gregory*, M.D. Part I. Inorganic Chemistry. 8vo, pp. 688. London.

3. A New, Universal, Etymological, and Pronouncing Dictionary of the English Language, embracing all the Terms used in Art, Science, and Literature. Parts 10, 11, 12, 13. Royal 8vo. London, Gilbert.

*This able and very useful work progresses with spirit.*

4. Gilbert's Modern Atlas of the World for the People, with an Introduction to the Physical Geography of the Globe, with an Alphabetical Index of 25,000 Places. Parts 8, 9, 10. Small Folio. London.

*Prepared with very great care, and distinguished for extreme accuracy. We can confidently recommend it.*

5. A System of Surgery. By *J. M. Chelius.* Translated from the German, and accompanied with additional Notes and Observations, by *John F. South.* Part 15. 8vo. London.

6. Clinical Facts and Reflections; also, Remarks on the Impunity of Murder in some Cases of presumed Insanity. By *Thomas Mayo*, M.D. 8vo, pp. 224. London.

7. The Medical Examiner and Record of Medical Science, Edited by *Robert M. Huston*, M.D. No. 19 and 20. New Series. July and August, 1846. Philadelphia.

8. Lectures on the Comparative Anatomy and Physiology of the Vertebrate Animals. Delivered at the Royal College of Surgeons of England in 1844 and 1846. By *Richard Owen*, F.R.S. Part I. Fishes. Illustrated with numerous Woodcuts. 8vo, pp. 319. London, 1846.

*In our next.*

9. The Retrospect of Medicine: being a Half-yearly Journal, containing a Retrospective View of every Discovery and practical Improvement in the Medical Sciences. Edited by *W. Braithwaite.* Vol. XIV. July to December, 1846. 8vo, pp. 466. London.

10. An Essay on the Tongue, in Functional Derangement of the Stomach and Bowels, and on the appropriate Treatment; also, the Tongue's Aspect in Organic Disease of the Lungs and Heart, &c. By *Edward Williams*, M.D., Cantab. &c. Second Edition. 8vo, pp. 236. London, 1846.

11. Practical Observations on some of the Diseases of the Stomach and Alimentary Canal. By *James Alderson*, M.D. 8vo, pp. 223. Plates. London, 1847.

12. Researches into the Physical History of Mankind. By *James Cowles Prichard*, M.D. Vol. V. Researches into the History of the Oceanic and of the American Nations. 8vo, pp. 585. Plates. London, 1847.

13. The American Journal of the Medical Sciences. Edited by *Isaac Hays*, M.D. Nos. 24 and 25. Oct. 1846. Jan. 1847. Philadelphia.

14. On the Correlation of Physical Forces; being the Substance of a Course of Lectures delivered in the London Institution in the Year 1843. By *W. R. Grove*, M.A., F.R.S. 8vo, pp. 52. London.

15. Experimental Researches on the Post-mortem Contractility of the Muscles, with Observations on the Reflex Theory. By *Bennet Dowler*, M.D. Re-printed from the New York Journal of Medicine for May. 8vo, pp. 39. New York.

16. Lecture Introductory to a Course of Clinical Medicine, delivered in the Theatre of Queen's College, Birmingham, on Tuesday, Dec. 1st, 1846. By *Samuel Wright*, M.D. 8vo, pp. 23. London.

*Well worthy the attention of the clinical student.*

17. The Half-yearly Abstract of the Medical Sciences; being a practical and analytical Digest of the Contents of the principal British and Continental Medical Works published in the preceding Six Months; together with a Series of Critical Reports on the Progress of Medicine and the Collateral Sciences during the same Period. Edited by *W. H. Ranking*, M.D. Vol. XIV. July—Dec. 1846. 8vo. pp. 492. London.

18. Body and Soul; or Life, Mind and Matter, considered as to their peculiar Nature and combined Condition in Living Things, with a View to render the Physiology of Life and Mind, more easily understood by the general Reader. Illustrated by Drawings. By *George Redford*, M.R.C.S. 8vo, pp. 242. London, 1847.

19. The Nature and Faculties of the Sympathetic Nerve. By *Joseph Swan.* 8vo, pp. 63. London, 1847.

20. An Inquiry into the Action of Mercury on the Living Body. By *Joseph Swan.* Third Edition. 8vo, pp. 34. London, 1847.

21. Letters to the Right Hon. Lord J. Russell, M.P., on the Drainage of the Metropolis, State of the Thames, and Waste of Fertilizing Matter; with an Appendix, containing Statements respecting the Impracticability of the Plan of the Metropolitan Sewerage Company, &c., and a Map of the proposed Works of the London Sewage Company. 8vo, pp. 16. London, 1847.

22. On Indigestion and certain Bilious Disorders often conjoined with it; to which are added, short Notes on Diet. By *George Chaplin Child*, M.D. 8vo, pp. 230. London, 1847.

23. A manual of the Principles and Practice of Ophthalmic Medicine and Surgery. By *J. Wharton Jones*, F.R.S. 8vo, pp. 606. London, 1847.

24. The London and Provincial Medical Directory, 1847. 8vo, pp. 568. London.

25. On Tumors of the Uterus and its Appendages. Jacksonian Prize Dissertation. By *Thomas Stafford Lee.* 8vo, pp. 290. London, 1847.

26. On the Mechanism of Respiration. By *Francis Sibson*, Esq. From the Philosophical Transactions. Part IV. for 1846. 4to, pp. 50. Plates. London, 1847.

27. Observations on the History and Treatment of Dysentery and its Combinations; with an Examination of their Claims to a Contagious Character, and an Inquiry into the Source of Contagion in its Analogous Diseases, Angina, Erysipelas, Hospital Gangrene, and Puerperal Fever. By *William Harty*, M.D. Second Edition. 8vo, pp. 323. London, 1847.

*We shall give an extended review of this excellent work in our next number. It is full of most interesting and valuable information.*

28. Medical Statistics, their Force and Fallacies. A Lecture delivered in Park Street School of Medicine. Nov. 4th, 1846. Introductory to the Course on the Theory and Practice of Physic. By *James F. Duncan*, M.D. 8vo, pp. 42. Dublin.

*Our notice is unavoidably deferred.*

29. Outlines of Structural and Physiological Botany. By *Arthur Henfrey*, F.L.S. Foolscap 8vo, pp. 261, with numerous Illustrations. London, 1847.

30. Janus. Zeitschrift fur Geschichte und Literatur der Medecin. Von Dr. *A. W. E. Henschel.* II. Band. I. Heft. Breslau, 1847.

*This journal is devoted, in a particular manner, to the literature and history of Medical Science. It is conducted by Dr. Henschel, with the aid of many of the most celebrated medical scholars in Germany and France; the only English name, appearing among his collaborateurs, is that of our distinguished friend, Dr. Greenhill of Oxford. The chief articles in the present number are, a paper by Dr. Ermerius of Groningen, on the Galenic Text of Hippocrates; one on the "Morbus Cardiacus" of the old Physicians; one on the distinguished Physicians and Surgeons of the 13th and 14th Centuries; and a most admirable one on Medical Reform, more especially in reference to medical education, by Dr. Carus. Had space permitted, we should have given some extracts from this last paper.*

31. Observations on Cancer. By *John Hughes Bennett*, M.D., F.R.S.E. *(From the Edinburgh Journal of Medical Science, for March,* 1847.)

32. On the Sanatory Condition of Newcastle-on-Tyne, and the Means necessary for its improvement; being a Lecture delivered before the Literary and Philosophical Society of that Town, on the 10th of February, 1847. By *George Robinson*, M.D. 8vo, pp. 58.

33. A Treatise on Diet and Regimen. By *William Henry Robertson*, M.D. 4th Edition, Part I. 8vo, pp. 124. London, 1847.

*In our next.*

34. Gray's Supplement to the Pharmacopœia; being a concise but comprehensive Dispensatory and Manual of Facts and Formulæ for the Chemist, Druggist, and Medical Practitioner. Entirely re-written, re-arranged, and considerably enlarged. By *Theophilus Redwood.* 8vo, pp. 1122. London, 1847.

*In our next.*

35. On Wounds and Injuries of the Abdomen and Pelvis; being the Second Part of the Lectures on some of the more important points in Surgery. By *G. J. Guthrie.* 8vo, pp. 73. London, 1847.

*In our next.*

36. The Royal Gazette of British Guiana, September 26th, 1846.

*This number contains some useful colonial hospital statistics, very creditable to Dr. Blair, their compiler.*

37. On Cataract, Artificial Pupil, and Strabismus. By *F. H. Brett*, M.D., F.R.C.S. 8vo, pp. 89, Woodcuts. London, 1847.

38. Copland's Dictionary of Practical Medicine. Part XI.

39. Revue Medicale, from August to Nov., 1846. *In exchange.*

40. Dublin Medical Review, No. 5.

*In exchange.*

41. Edinburgh Medical and Surgical Journal, for January.

*In exchange.*

42. British and Foreign Medical Review, for January.

*In exchange.*

43. Edinburgh Monthly Journal of Medical Science, for January, February, and March.

*In exchange.*

44. L'Union Medicale. January to April.

*In exchange.*

45. Medical Gazette, January to April.

*In exchange.*

46. Gazette Medicale, January to April.

*In exchange.*

---

*Want of room compels us to omit the Selections from Foreign Journals in our present number.*

### INDEX.

Abscess, communicating with aortal arch .......... 339
Acid, Prussic, poisoning by ........ 115
Acid, sulphuric, poisoning by ...... 113
Addison on auscultation .......... 90
Ægineta, Paulus, writings of........ 311
Ætius, writings of................ 311
Albuminuria, mercury in .......... 114
Alcohol as a respiratory element .... 140
Alderson on diseases of the stomach.. 537
Alexander of Tralles .............. 311
Alexandrian School, the......... .. 298
Algeria, meteorology of........... 79
Algeria, diseases of................ 81
Amaurosis, spectacles in .......... 202
Amblyopia from presbyopia ........ 201
Amenorrhœa, secondary .......... 52
Amputation by the flap............ 71
Anatomy at Alexandria............ 299
Anatomy, under Hippocrates........ 293
Anatomy, progress of..... ........ 403
Anatomy, pathological, Vogel's...... 429
Anæmia, disorder of heart's action in 343
Aneurism, femoral................. 318
Angina pectoris, cases of .......... 355
Angina pectoris, treatment of ...... 360
Animism, theory of .......... .. 421-7
Aorta, inflammation of the ........ 368
Aperients for children ............ 124
Arabs, condition of medicine under.. 314
Arachnoid, ganglionic character of the 320
Aretæus, writings of .............. 301
Aristotle, influence of on medical science...................... 297
Arsenic, poisoning by.............. 114
Arteries, condition of, in disease of the heart ...................... 46
Arteries, Bouillaud on inflammation of 366
Arteries, Liston on wounds of ...... 322
Artery, subclavian, ligature of ...... 228
Artery, carotid, ligature of ........ 230
Artery, posterior tibial, ligature of .. 232
Artery, pulmonary, inflammation of.. 369
Artery, brachial, wounds of ....... 552
Assafœtida, description of.......... 238
Astragalus, dislocation of .......... 102
Auscultation, fallacies of .......... 90
Avicenna, writings of.............. 315
Baglivi, the opponent of humorism.. 426
Bebeerine, account of.............. 240
Behr, Handbook of Anatomy ...... 254
Bile, Liebig on origin and uses of.... 139
Bladder, rupture of the ........101, 554
Blood, condition of, in fever ........ 85
Blood, Hewson and Jones on...... . 1
Blood, condition of, in inflammation 7
Blood, coagulation of the .......... 9
Blood, red corpuscles of..........10, 17
Blood of the embryo.............. 14
Blood-letting, Hall on ............ 162
Boerhaave, biographical sketch of.... 172
Boerhaave, Marx' letter to ........ 176
Boerhaave, influence and talent of .. 422
Boerhaave, Institutiones of ........ 426
Borelli on animal mechanics........ 426
Botany, mode of study of.......... 370
Botany, influence of the cell-theory on the study of.................... 372
Botany, natural system of.......... 379
Bouillaud, Nosographie Medicale.. . 341
Brain, disease of, after ligature of the carotids ...................... 230
Brain, localization of the faculties of 154
Bread, unfermented .............. 146
Breast, cancer of.................. 112
Brett on cataract ................ 527
Bright's disease, pathology of....216, 222
Brown's Theory of disease.......... 428
Bursæ, treatment of enlarged ...... 70
Bushnan on hydropathy .......... 245
Cancer, Vogel on development of .... 449
Cancer of the stomach ............ 538
Cannabis sativa .................. 241
Carminatives for children .......... 127
Carpenter's Physiology ............ 254
Cataract, operation for ............ 527
Cell-development, theory of ....372, 437
Cell-development, agency of endosmose in ...................... 513
Chauliac, Gui de .............. .. 401
Chemistry, relation to physiology.... 146
Cheyne, biographical sketch of .... 183
Cheyne, Marx' letter to .......... 189
Children, regulation of the sleep of.. 122
Children, importance of country air for 123
Children, milk regimen in diseases of 123
Children, effects of remedies upon .. 124
Children, diet of.................. 145
Childs on Indigestion.............. 550
Chlorosis, disorder of the heart's action in........................ 343
Cholera, remedy in the ............ 252
Cholera, Copland on ..........460, 494
Cholera, spasmodic and pestilential .. 463
Cholera, history of progress 467-81-89-91
Cholera, question of infection ..471, 497
Cholera, Indian reports on.......... 473
Cholera, progress of in England .... 485
Chorea, Hughes on .............. 109
Chorea, connection with rheumatism 109
Chorea, treatment of .............. 110
Circulation, history of discovery of the 411

Coction, Hippocractic doctrine of.... 295
Conjunctiva, congenital tumour of .. 526
Contagion,chemical and parasitic theories ....................149, 373
Contagion and infection distinguished 457
Cooper on near-sight........ ..... 196
Copland's dictionary of medicine .... 456
Corpus callosum, absence of the .... 317
Cranium, discharge of fluid from the ears in fracture of ............. 70
Critical days, Hippocratic doctrine of 295
Cullen, the nosology of............ 419
Cullen's theory of disease.......... 429
Cyanosis, case of ............. .. 319
Cytoblastema, development of the .. 437
Davies on diseases of children...... 117
Death, sudden, Lombard on........ 272
Dentition, paralysis during ........ 155
Development, effects of the arrest of.. 453
Diarrhœa of Algeria .............. 82
Diarrhœa of children.............. 123
Dietetics, importance of ...... 189
Diseases, new, discovery of ...... .. 407
Dogmatism of the ancients ........ 295
Dogmatism, influence of Aristotle upon 297
Dogmatism in medicine............ 302
Dowler on the reflex theory ........ 546
Dropsy from cardiac disease ........ 355
Dropsy, nature and varieties of .... 432
Dropsy, ovarian, treatment of ...... 508
Dysentery in connexion with hepatic abscess ...................... 255
Dysentery, treatment of............ 256
Dysmennorrhœa.................. 52
Ear, foreign bodies in the.......... 87
Eclectism, doctrine of ........... 309
Education, defective popular........ 168
Electricity, muscular currents of .... 515
Elevation in surgical cases ........ 275
Ellis on clinical surgery............ 552
Emetics for children .............. 126
Emetics, Royle on ................ 243
Empiricism doctrines and history of 306
Empiricism, revival of rational .... 428
Endosmose, discovery of .......... 511
Endosmose, agency on cell-life...... 513
Endosmose, Matteucci's experiments 514
Endocarditis, Latham & Bouillaud, 35, 361
Enemata, administration of food by.. 73
Enemata for children.............. 125
Epigenesis, pathological............ 435
Eruptions, venereal, treatment of.... 74
Erysipelas, infantile .............. 128
Erysipelas, epidemic .............. 279
Ether, inhalation of, considered .... 530
Experiments upon animals ......156, 161
Eyes, accommodation of, to distances 197
Eyes, preservation of the .......... 204
Eyes, luminosity of the............ 336
Femur, Velpeau on fracture of cervix 270
Fernel, pathological doctrines of .... 404
Fevers, Algerian.................. 83
Fevers, Algerian, analysis of blood in 85
Fevers, eruptive, described by the Arabs 315
Fevers, Mayo on petechial ........ 386
Fever, softening of the heart in...... 351
Fever, intestinal depositions in...... 445
Fibula, fracture of the ............ 69
Food, influence of variety of, on man 143
Food, proportions of nutriment in, 144, 340
Foramen Botalii, patency of ........ 388
Forceps, midwifery, statistics of .... 65
Frog, electric currents of .. ....... 516
Galen, doctrines of................ 302
Galen, influence of the Platonic philosophy on...................... 305
Generative system, views of ancients on 414
Glands, Hewson on structure of .... 20
Glover on scrofula............ ... 132
Greek commentators, the .......... 510
Griffiths' chemistry of the four seasons 248
Guy's Hospital Reports............ 90
Hæmorrhage, uterine, varieties of .. 59
Hæmorrhage, uterine, treatment of .. 62
Hæmorrhage, uterine, Radford on unavoidable .................... 63
Hæmatocele, spontaneous.......... 72
Hair, structural anatomy of ........ 210
Hall's, Dr. M., Observations........ 151
Hallé, biographical sketch of........ 191
Hallé, Marx' letter to ............ 192
Haller, physiology of.............. 418
Harden on isopathia .............. 259
Hassall, microscopic anatomy ..253, 547
Health, the proposed officers of...... 25
Heart, auscultation of the......... 97
Heart, prognosis in disease of the .. 33
Heart, secondary inflammation of, 35, 361
Heart, laceration and suppuration of the...................... 40, 366
Heart, shocks of the .............. 41
Heart, rupture of the valves of during effort ............ .......... 42
Heart, treatment of valvular disease of 46
Heart, complication of valvular disease and hypertrophy of......348, 365
Heart, diagnosis, causes and treatment of hypertrophy of the ...... 46, 343-7
Heart, aneurism of the............ 366
Heart, disease of the, a cause of sudden death . ..................... 272
Heart, chronic disease of, altered conditions of the blood in .......... 349
Heart, atrophy of the.............. 351
Heart, chronic consecutive lesions of 352
Henfrey's structural botany ........ 383
Hernia, pseudo, strangulation of .... 26
Hernia, humoralis, treatment ...... 76
Hewson, biographical sketch of...... 1
Hewson, observations on the blood .. 5
Hippocrates, state of medicine in his time........................ 292
Hippocrates, influence of the Socratic philosophy upon................ 296

Hippocrates, aphorisms of.......... 294
Hoffman's Theory of Medicine ...... 428
Hospitals, origin of ............. 313
Hospitals, leper, of the middle ages.. 402
Hufeland, the relations of physicians 548
Hunter, John, merits of............ 421
Hutchinson on the spirometer ...... 328
Hydrocephaloid disease............ 130
Hysteria, Mayo on local............ 390
Ileus, cure of ................... 387
Indigestion, Childs on ............ 550
Infants, ophthalmia of ............ 524
Infection, definition of ............ 457
Inflammation, nature of............ 157
Insanity, irrigation in ............ 281
Intemperance, effects in hospitals.... 70
Intestinal canal an organ of secretion 142
Iodine, employment in syphilis .. 77, 267
Iodine in scrofula ................ 137
Iron, administration of, to children.. 128
Iron, the sulphate of .............. 237
Irritability, Haller on.............. 415
Jacob on ophthalmia............... 520
Jacquemier, Manuel des Accoucheurs, 49
Jones, Wharton, on the blood corpuscle 1
Jones, Wharton, on ophth. medicine 518
Kennedy on Epidemic Cholera ...... 252
Kidney, pathology of.............. 216
Kidney, granular degeneration of 225, 252
Labour, induction of premature .... 68
Language, localization of faculty of.. 86
Laryngismus stridulus ............ 129
Latham on diseases of the heart .. 33, 341
Lead, poisoning by............ 115, 270
Lee on tumour of the uterus........ 498
Lee on mineral waters ............ 555
Leguminosæ, poisonous species of .. 381
Lichen, pathology of .............. 213
Liebig's animal chemistry.......... 138
Light, varieties of artificial ........ 207
Linacre, great influence of ........ 402
Lindley's Vegetable Kingdom ...... 370
Liston on wounded arteries ....... 322
Little on diseases of the eye ........ 196
Liver, abscess of the .............. 83
Liver, minute anatomy of .......... 106
Lombard on sudden death .......... 272
Lungs, capacity of ................ 328
Lungs, condition of, in heart disease 353
Lymphatics, discovery of .......... 419
Mackness' Moral Aspects of Medical Life ......................... 377
Maize, localities of................. 377
Malformations, nature and classification of......................... 452
Malgaigne's History of Surgery .... 285
Malgaigne on hernia .............. 260
Materia Medica, Royle's............ 234
Matteuci's Lectures and Researches.. 509
Mayo's Clinical Facts ............ 385
Mead, Biographical Sketch of ...... 179
Mead, Marx' letter to ............ 181
Mechanical theory of physic ...... 425
Medical science, progress of 292-8, 310-14
Medico-Chirurgical Transactions 216, 317
Medicine, Renouard's history of, 285, 399
Medicine, Greek, period of.......... 311
Medicine, progressive organization of the practice of ............ 312, 399
Medicine, reformatory period ...... 410
Medicine, accessory institutions to .. 313
Medicine, mechanics and chemistry applied to .................... 425
Medicines new, introduction of .... 410
Medicine, the various theories of.... 414
Melanosis, Vogel on .............. 440
Menstruation, disorders of ......... 52
Mesmerism, Mayo on............... 393
Mercury, employment in syphilis.. 73, 77
Mercury, salivation by small doses of, 113
Mercury, employment in inflammation 276
Mercury, Swan on action of........ 549
Metamorphoses, vegetable ........ 374
Methodism in medicine............ 308
Milk, condition prior to delivery .... 117
Milk, composition of ..........119, 144
Milk, regimen of for diseased children 123
Milk, influence of articles of food in.. 143
Milk, adaptation for food ..........
Monstrosity, partial double ........ 321
Moore on the relation of body and soul 249
Morgagni, pathological letters of ... 417
Morphology ..................... 374
Mortality in England and Europe.... 169
Mulder's reply to Liebig .......... 247
Muscle, electrical current of........ 515
Muscle, induced contraction of ..... 517
Muscle, post-mortem contractility of, 544
Myopia, acquired ................ 203
Myopia, Cooper on ................ 205
Nails, semeiological value of........ 257
Narcotics for children ............. 126
Negrier on the cervix uteri ......... 49
Nerve, the 5th, tumour in the ...... 327
Nervous system, progress of discovery, 413
Nodes, treatment of .............. 76
Nosography, progress of.. 293, 300, 419
Nurses, Donné on requisites of ..... 118
Observation, Liebig on ............ 147
Ophthalmia, observations on ....... 518
Ophthalmia, scrofulous ........... 520
Ophthalmia, rheumatic............. 521
Ophthalmia, gonorrhœal .......... 522
Ophthalmia, infantile .............. 524
Oribasius, writings of ............. 311
Ormerod's clinical collections ...... 67
Ovariotomy, Lee on ............... 505
Ovary, encysted dropsy of.......... 502
Paracelsus, estimate of............. 409
Parasites, question of origin of...... 449
Paré, Ambrose, account of ........ 406
Pathological epigenesis ........... 435
Pathology, progress of ....293, 300, 404
Pathology, mode of investigating.... 430

Pericarditis, prognosis ............ 33
Pericarditis, secondary ............ 35
Pericarditis, Bouillaud on .......... 361
Pericardium, inflammatory adhesion of 39
Periodicity of disease.............. 389
Periostitis, syphilitic ............. 76
Pertussis, alum in ................ 130
Pestilence, nature of .............. 456
Phlebitis, cases of ............ 100, 103
Phthisis, influence of pregnancy on .. 57
Phthisis, immunity from in Algeria.. 24
Phthisis, diagnosis of, obscured by bronchitis ................... 92
Physiology, relations of chemistry to, 146
Physiology, influence of anatomy in.. 150
Physiology, progress of vegetable.... 375
Pinel, Philip, nosography of........ 419
Placenta presentation .......... 59, 63
Plants, Schleiden on the sexes of .... 375
Pleurisy seated low down .......... 96
Pneumonia, influence of pregnancy in 95
Pneumonia, latent................. 277
Pneumonia, seated at the apex...... 95
Porrigo, treatment of.............. 131
Porrigo, pathology of .............. 214
Potatoe disease, Smee on .......... 251
Pregnancy, bleeding during ........ 54
Pregnancy, influence of disease on .. 55
Presbyopia, Cooper and Sichel on ... 199
Prostate, hydatids of the .......... 333
Protein, Mulder on ................ 247
Prurigo, Wilson on................. 213
Ptosis, France on ................ 98
Puerperal disease, Hall on ......... 158
Pylorus, carcinoma of ............. 540
Redford on body and soul.......... 548
Registrar-general's report.......... 166
Renouard histoire de la medecine.... 285
Respiration an indirect combustion.. 141
Respiration, elements of ........... 141
Respiration, Hutchinson on ......... 328
Respiration, Sibson on ............. 560
Respiration, history of ............ 412
Rhazes, writings of ............... 314
Rheumatism in pregnancy ......... 56
Robinson on inhalation of ether .... 530
Royle on materia medica .......... 234
Sacrum, dislocation of ............ 103
Salerno, school of .. ............. 399
Sanctorius, experiments of ........ 416
Sanitary Bill ..................... 27
Scarlatina, treatment of ........... 391
Scrofula, Glover on the nature of ... 133
Secale cornutum, powers of the .... 57
Sedatives for children............. 126
Sewers, fertilizing power of ........ 31
Sight, aged ...................... 199
Sight, short....................... 205
Sirocco, effects of the ............ 80
Skin, papular disease of the ........ 212
Sleep, Hall on ................... 154
Sleep of children ................. 122
Small-pox, blisters in ............. 258
Smee on the potatoe disease ........ 251
Spectacles, Sichel on .............. 197
Stahl's system of medicine ......... 427
Stimulants for children............ 127
Stomach, perforation of the ........ 107
Stomach, Alderson on diseases of the, 537
Stomach, carcinoma of the ........ 538
Stomach, hypertrophy of coats of the, 543
Strabismus, Brett on.............. 529
Suckling, indications of power for .. 117
Surgery, progress of ..........405, 420
Swan on mercury ................ 549
Sydenham, clinical observations of .. 423
Syphilis, iodine treatment of........ 267
Syphilis, Ormerod on.............. 73
Thomson on food of animals.... 138, 339
Throat, syphilitic ulcers of .......... 75
Thumb, dislocation of the.......... 69
Tongue, Williams on the .......... 560
Tonics for children ............... 127
Towns, health of .................. 21
Trephine, Ellis on the ............ 554
Trichosis furfuracea .............. 216
Tubercle, nature of............132, 446
Tumours of the Uterus, Lee on...... 498
Tumours, Vogel's classification...... 441
Tumours, encysted ............... 447
Tumours, development of malignant, 448
Turnbull on physical diagnosis...... 256
Typhus, glandular deposits in ...... 445
Ulcers, treatment by elevation ...... 275
Underwood, diseases of children .... 117
Universities, origin of ............ 400
Urethra, varicose tumour in the female 105
Urethritis, diluents injurious in...... 269
Uterus, peculiarities of cervix ...... 50
Uterus, watery discharge from ...... 54
Uterus, fungoid disease of ......104, 502
Uterus, operation for scirrhus ...... 105
Uterus, ulceration of cervix ........ 105
Uterus, granular cervix of.......... 263
Uterus, influence of diseases of, on the position of the child ........... 262
Uterus, fibrous tumours of ........ 498
Uterus, cauliflower excrescence of .. 502
Valves, cardiac, rupture of the...... 42
Varicocele, pressure in ............ 333
Veins, murmurs in, in chlorosis .... 345
Version, cephalic ................ 58
Vesalius, Andrew ................ 403
Vision, impaired, Cooper on ....... 207
Vital forces, operation of the ...... 415
Vogel's pathological anatomy ...... 429
Waters, mineral, Lee on .......... 556
Williams on the tongue............ 550
Willis' system of medicine ......... 425
Wilson on diseases of the skin ...... 209
Worms, treatment of.............. 129

